# Imaging Manifestations of Creutzfeldt-Jakob Disease and Case Series

**DOI:** 10.7759/cureus.3725

**Published:** 2018-12-13

**Authors:** David R Warden, John V Dennison, Joseph Limback, Seema M Shroff, Steven A Messina

**Affiliations:** 1 Radiology, Florida Hospital-Orlando, Orlando, USA; 2 Pathology, Florida Hospital-Orlando, Orlando, USA

**Keywords:** creutzfeldt-jakob disease (cjd), sporadic creutzfeldt-jakob disease, cjd, prion

## Abstract

Sporadic Creutzfeldt-Jakob disease (CJD) is the most common prion disease, resulting in rapid neurocognitive decline, and is universally lethal. CJD has a confounding clinical presentation with similarities which overlap with many other neurodegenerative disorders. Brain biopsy is the current gold standard; however, less-invasive initial screening tests are also utilized. These include brain magnetic resonance imaging (MRI), electroencephalography (EEG), and cerebrospinal fluid (CSF) laboratory studies.

Five patients presented to our facility with varying levels of nonspecific cognitive impairment and movement disorders. CJD was initially suggested after review of each patient’s brain MRI. The T2-weighted fluid attenuation inversion recovery and diffusion-weighted images in each case demonstrated varied classic patterns of signal abnormality involving the cortex, basal ganglia, thalami, and brainstem. EEG and CSF studies were confirmatory in three and four patients, respectively (EEG not performed in one patient). One death occurred two months after initial presentation, and the other four patients were transferred to hospice three, four, nine, and 20 months after initial presentation.

Radiological evaluation is an invaluable component of the workup for nonspecific neurodegenerative disorders because brain MRI may suggest the initial diagnosis of CJD, as demonstrated in our presented cases. Familiarity with the spectrum of classic MRI findings suggestive of sporadic CJD can improve radiologists’ role in early detection of the most common prion disease. Clinicians may benefit from understanding the utility of the newer CSF laboratory studies (Real-time quaking-induced conversion, T-tau, and 14-3-3 protein), which are far less invasive than the gold standard of brain biopsy. Early diagnosis can help save medical resources and guide clinicians to form appropriate plans of care with the patient and family.

## Introduction

Creutzfeldt-Jakob disease (CJD) is an uncommon cause of dementia, but must be considered as a differential diagnosis when suggested by a rapid onset, suggestive electroencephalography (EEG), or imaging findings. CJD has a global incidence of approximately 0.5 to one cases per million [1]. There are three types of CJD: sporadic (85% of cases), familial, or acquired (kuru, iatrogenic, bovine spongiform encephalopathy/variant CJD) [2]. A case series in Thailand demonstrated an average age of onset in sporadic CJD of 60 years, and the average length of the illness was four months. Infection, heart failure, and respiratory arrest account for the majority of mortality in affected in patients [3].

CJD is a prion disease, which demonstrates unique findings on pathologic analysis. Gray matter undergoes neuronal loss and vacuoles form in the neuropil creating a “sponge-like” appearance [3]. Newer cerebrospinal fluid (CSF) laboratory studies, such as real-time quaking-induced conversion (RT-QuIC), T-tau, and 14-3-3 protein, provide a less invasive alternative to diagnosis compared to the gold standard of brain biopsy. Findings on magnetic resonance imaging (MRI) can have a variable distribution of increased signal on T2-weighted fluid attenuation inversion recovery (T2-FLAIR) imaging and diffusion-weighted imaging (DWI) with associated low apparent diffusion coefficient (ADC) values.

## Case presentation

Five cases of CJD at our institution are discussed below with a review of clinical, imaging, and laboratory findings. Based on MRI findings, the diagnosis of CJD can be suggested by University of California-San Francisco (UCSF) revised criteria proposed in 2011, summarized in Table 1 [4]. The UCSF criteria outline specific sites in the brain for the radiologist to analyze on DWI and T2-FLAIR images to subsequently provide the clinician with a categorization of “MRI definitely CJD”, “MRI probably CJD”, “MRI definitely not CJD”, or “Other MRI issues” (Table 1). Imaging of these five patient cases is reviewed below using the 2011 UCSF modified grading system, which illustrate the full spectrum of expected signal abnormalities on MRI associated with CJD.

**Table 1:** Proposed University of California-San Francisco (UCSF) 2011 revised criteria*. 1. Supportive for cortical involvement: Asymmetric involvement of midline neocortex or cingulate *or* sparing of the precentral gyrus *or* ADC cortical ribboning with low values. 2. Supportive for subcortical involvement: Striatum with anterior-posterior gradient *or* subcortical low ADC values. *Adapted from original table of proposed criteria [4]. DWI: Diffusion-weighted image; T2-FLAIR: T2-weighted fluid attenuation inversion recovery; MRI: Magnetic resonance imaging; CJD: Creutzfeldt-Jakob disease; ADC: Apparent diffusion coefficient.

Diagnosis	Criteria
MRI definitely CJD	DWI > T2-FLAIR hyperintensity in:
	Classic pathognomonic: cingulate, striatum, and >1 neocortical gyrus (often precuneus, angular, or superior/frontal gyrus)
	Cortex only involvement (>3 gyri); see supportive for cortical^1^
MRI probably CJD	Unilateral striatum or cortex (≤3 gyri); see supportive for cortical and subcortical^1,2^
	Bilateral striatum or posteromesial thalamus; see supportive for subcortical^2^
MRI probably not CJD	Only T2-FLAIR/DWI abnormalities in limbic areas, where hyperintensity can be normal (e.g., insula, anterior cingulate, hippocampi) and ADC map does not show restricted diffusion in these areas
	DWI hyperintensities due to artifact (signal distortion); see other MRI issues (below)
	T2-FLAIR > DWI hyperintensities; see other MRI issues (below)
MRI definitely not CJD	Normal imaging
	Abnormalities not consistent with CJD
Other MRI issues	In prolonged courses of CJD (>1 year) brain MRI might show significant atrophy with loss of DWI hyperintensity, particularly in areas previously with restricted diffusion
	To help distinguish abnormality from artifact, obtain sequences in multiple directions (e.g., axial and coronal)

Case 1

A 67-year-old female initially presented with cognitive decline over two weeks, accompanied by vomiting, hallucinations, and blurred vision. She had a history of breast cancer and a recent cruise to the Bahamas, during which she remained on the boat due to inclement weather. She was admitted at an outside hospital and became progressively less responsive. She then became unresponsive and was transferred to our facility for critical care and video EEG monitoring.

**Figure 1:**
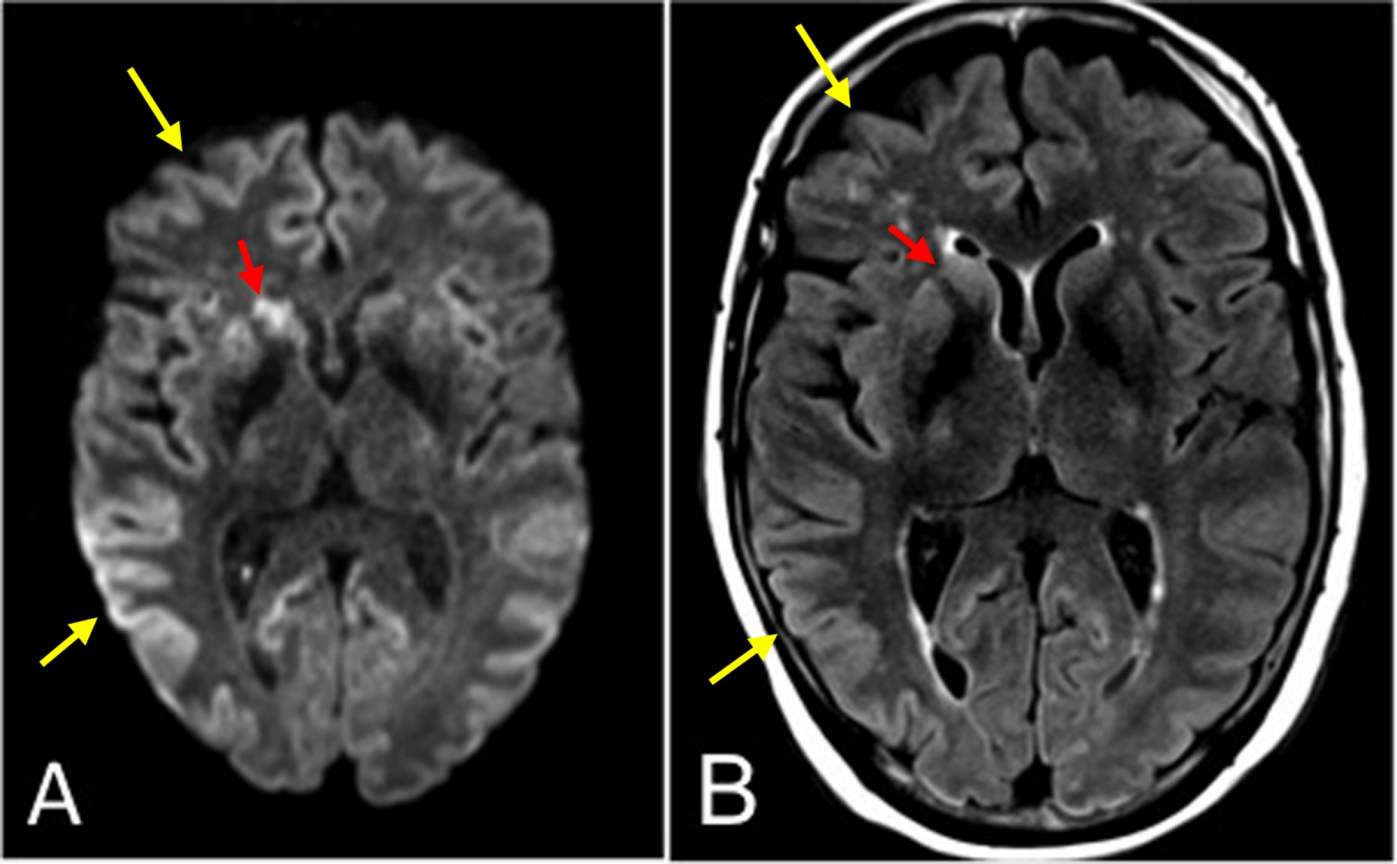
Axial magnetic resonance imaging (MRI) of Case 1. (A) Diffusion-weighted image (DWI) at the level of the basal ganglia demonstrating asymmetrically increased signal in the entire neocortex (yellow arrows) and striatum (red arrows). (B) T2-weighted fluid attenuation inversion recovery (T2-FLAIR) image with less corresponding hyperintense signal.

An MRI demonstrated asymmetric, diffuse hyperintensity of the cortex and striatum on T2-FLAIR and DWI sequences (Figure 1). Using the 2011 UCSF modified grading system, this case met criteria for “MRI definitely CJD.” Additional brain MRIs performed over the next three weeks showed rapidly progressive signal change on DWI. An EEG was performed demonstrating slowing in the theta frequency and generalized epileptiform discharges at a frequency of 1 Hz. Approximately one-half to two-thirds of patients with sporadic CJD demonstrate triphasic, biphasic, or mixed periodic sharp wave complexes at a rate of 1 Hz, typically at a later stage [5, 6]. Lumbar puncture testing of CSF yielded positive results of RT-QuiC, T-tau, and 14-3-3 protein. RT-QuiC has sensitivity and specificity of 87-91% and 98-100%, respectively [7]. The 14-3-3 and T-tau test combined have sensitivity and specificity of 79% and 99%, respectively [1]. The patient continued to deteriorate clinically and died two months after her symptoms began.

Case 2

**Figure 2:**
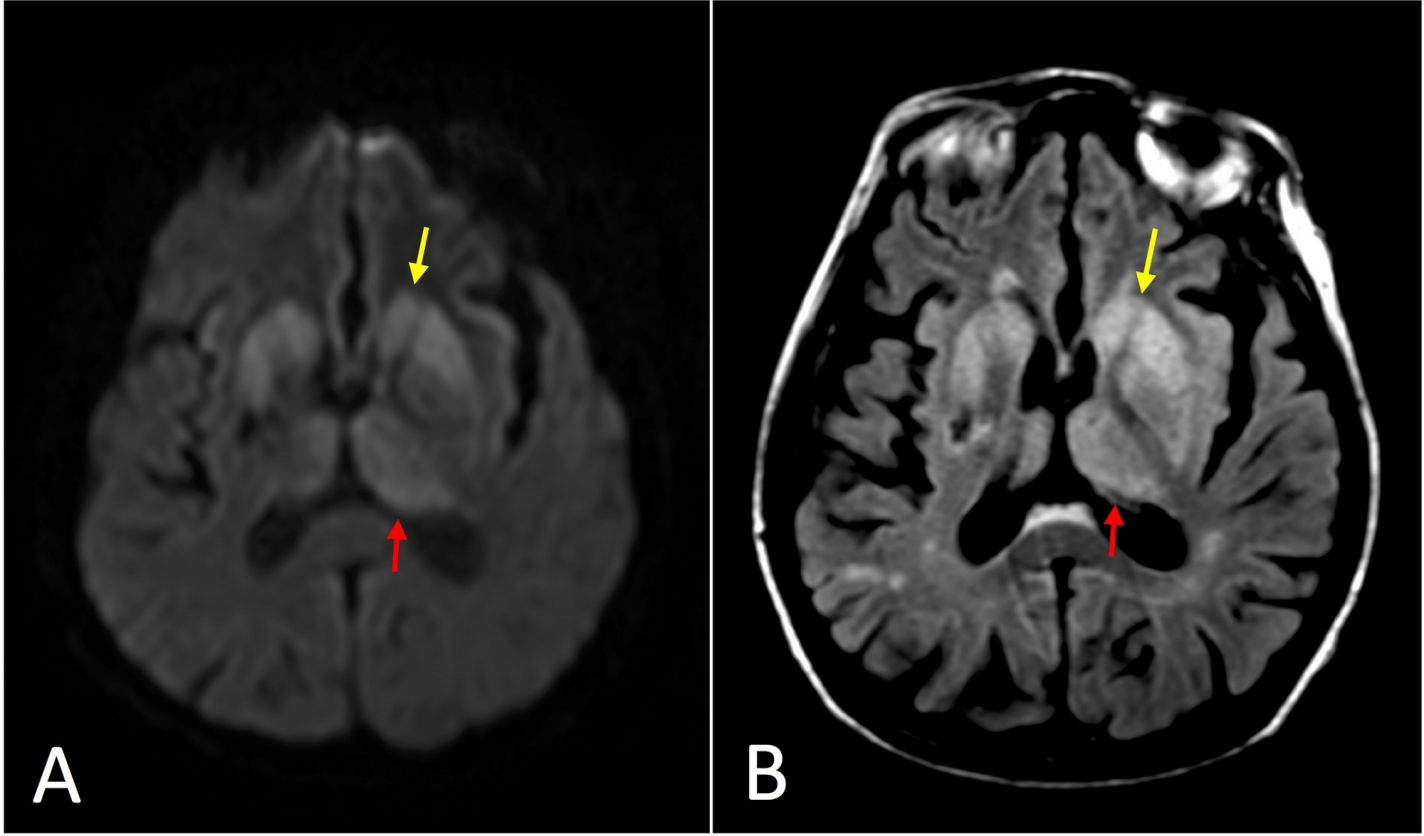
Axial magnetic resonance imaging (MRI) of Case 2. (A) Diffusion-weighted image (DWI) at the level of the basal ganglia demonstrating asymmetrically increased signal primarily in the striatum (yellow arrow) and medial and posterior thalamus (red arrow) reminiscent of the “hockey stick” sign frequently seen in variant Creutzfeldt-Jakob disease (CJD). (B) T2-weighted fluid attenuation inversion recovery (T2-FLAIR) image with less corresponding hyperintense signal.

A 61-year-old female with history of type 2 diabetes mellitus, hypertension, and hypothyroidism, presented with persistent dizziness. A brain MRI demonstrated an acute right middle cerebral artery infarct affecting the right insular cortex and right corona radiata without any evidence of CJD (“MRI definitely not CJD”). The patient was discharged after appropriate treatment for stroke. Six weeks later she presented from home with increasing confusion, weakness, difficulty ambulating, and hallucinations. A brain MRI demonstrated very subtle restricted diffusion in the bilateral frontal cortices, basal ganglia and thalami, greater on the left (Figure 2). This was interpreted as global hypoxic ischemia, and neurology started the patient on aspirin and Plavix. The patient was discharged to a nursing home. Using the 2011 UCSF modified grading system, the imaging met criteria for “MRI definitely CJD.”

One month later she was readmitted for acutely decreased responsiveness overnight. She underwent an initial stroke workup, and a third MRI was performed demonstrating progressive DWI hyperintensity in the caudate nuclei, lentiform nuclei, thalami, hippocampi, dorsal brainstem, and frontal and insular cortices. CJD was suggested by MR imaging, so the patient underwent lumbar puncture. An EEG demonstrated overall background activity in the 4 to 5 Hz delta range. Lumbar puncture testing of CSF eventually yielded positive results of RT-QuiC, T-tau, and 14-3-3 protein. The patient never improved during her hospital stay and went into cardiopulmonary arrest approximately four weeks later. She was intubated and resuscitated. Shortly afterward, the decision was made to withdraw care, and the patient was transferred to hospice four months after the initial presentation.

Case 3

**Figure 3:**
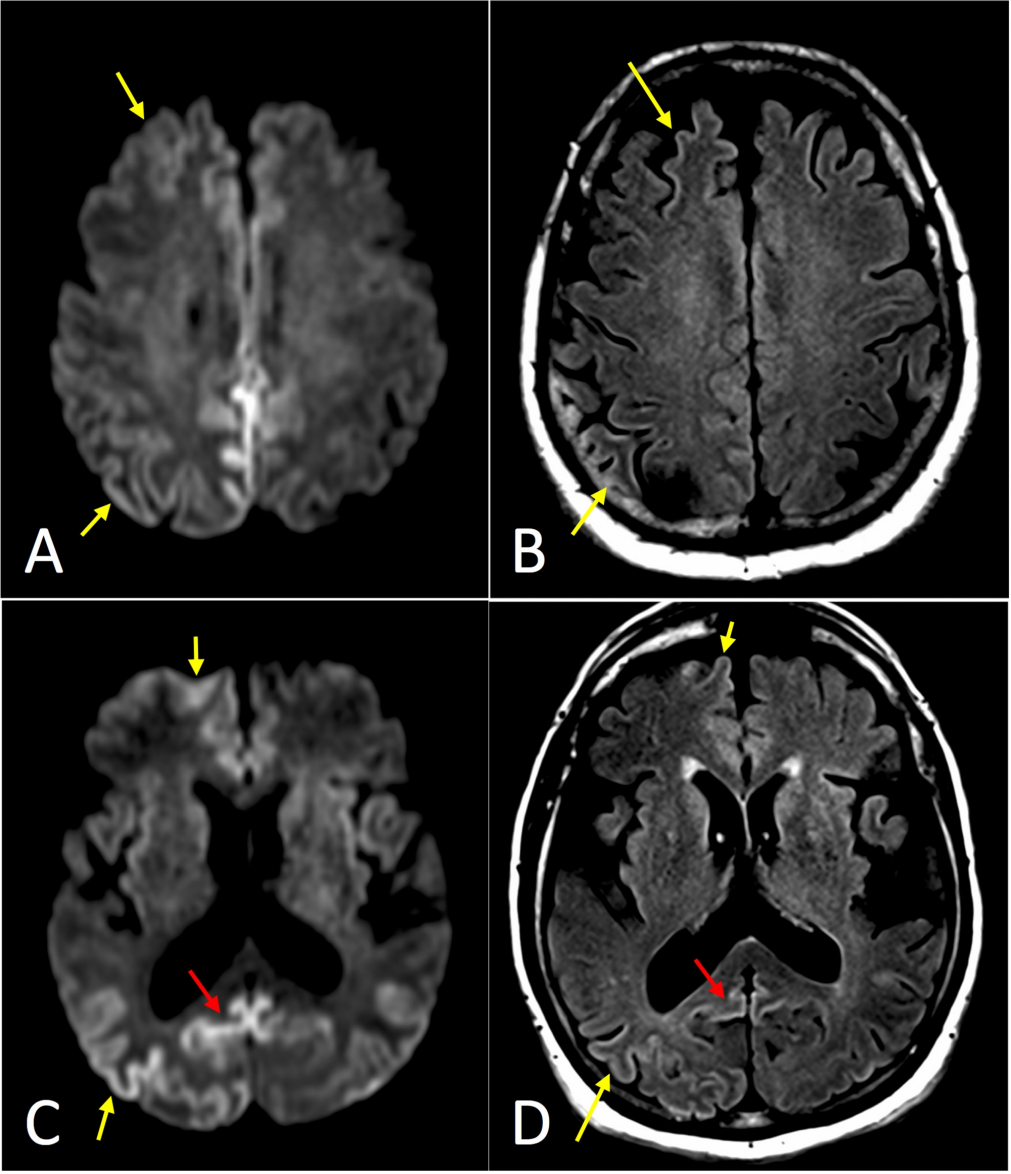
Axial magnetic resonance imaging (MRI) of Case 3. (A) Diffusion-weighted image (DWI) at the level of the centrum semiovale demonstrating asymmetrically increased signal in the frontal and parietal cortex (yellow arrows), with notable involvement of the cingulate gyrus. (B) T2-weighted fluid attenuation inversion recovery (T2-FLAIR) image with less corresponding hyperintense signal. (C) DWI at the level of the basal ganglia demonstrating asymmetrically increased signal in the neocortex (yellow arrows) and striatum, with notable involvement of precuneus (red arrow). (D) T2-FLAIR image with less corresponding hyperintense signal.

A 76-year-old male with history of prostate cancer initially presented to a primary care physician with slowly progressive confusion and memory loss. An MRI was obtained within our hospital network, which demonstrated asymmetric restricted diffusion involving the bilateral cortex and striatum with notable involvement of the precuneus (Figure 3). CJD was suggested as the top differential diagnosis in the radiology report, and the patient was sent to our facility for admission and additional evaluation. Using the 2011 UCSF modified grading system, the imaging met criteria for “MRI definitely CJD.” An EEG was normal. A lumbar puncture was performed, and the patient was discharged to follow up as an outpatient.

The initial CSF studies showed a positive RT-QuIC but negative T-tau and 14-3-3 protein results. Two months after the initial presentation, the patient went to UCSF, and the diagnosis of CJD was confirmed. The patient was treated with Niagen and Hismanal (astemizole). Six months after the initial diagnosis, the patient was reported to have intermittent confusion, gait disturbance, and occasional bladder and bowel incontinence. At this time he began receiving home hospice services (nine months after initial presentation). One year after the initial presentation, the patient had lost 40 lbs in the last six months, was nearly unresponsive, and unable to ambulate.

Case 4

A 72-year-old female presented with rapidly increasing dementia for the past two months, aphasia, hallucinations, and oculomotor and facial apraxia. Twenty months prior, the patient presented to an outside facility with dementia and gait disturbance and was diagnosed with Parkinson’s dementia. She demonstrated some initial improvement with carbidopa/levodopa, but the effect was only temporary. Eleven months prior to presentation at our institution, she underwent nasal surgery and afterward developed increasing confusion and hallucinations. Nine months prior to presentation, she had a fall and developed epidural and subdural hematomas, which confounded the clinical and imaging findings.

**Figure 4:**
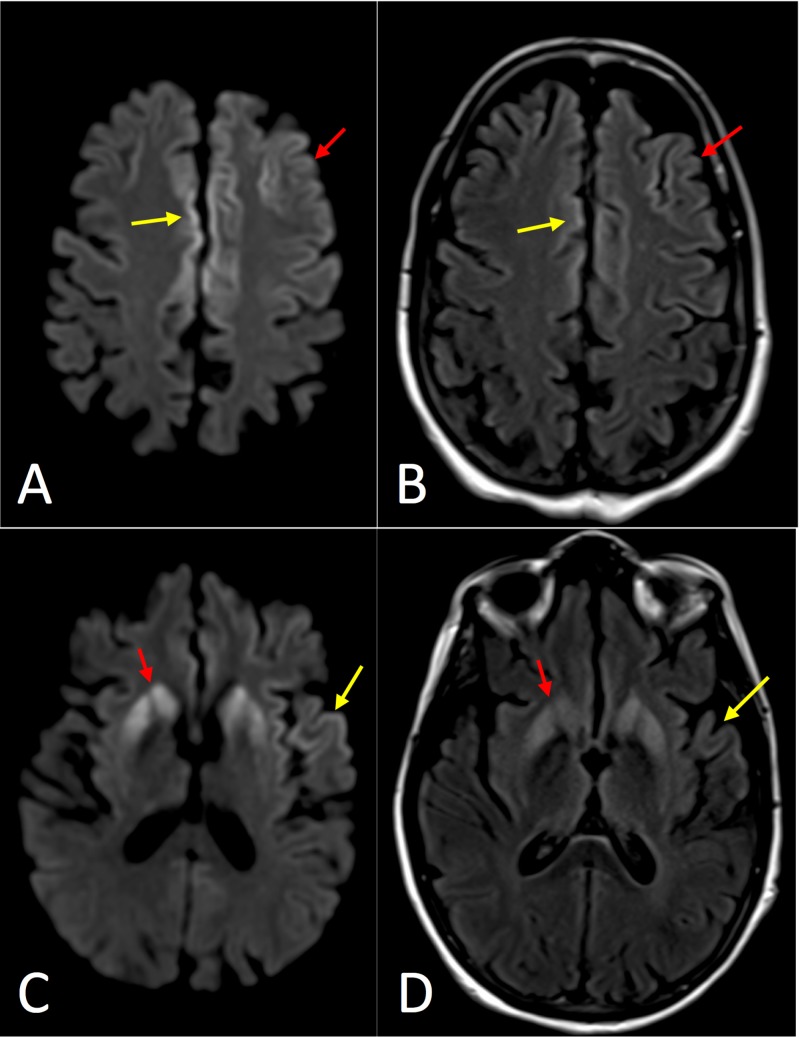
Axial magnetic resonance imaging (MRI) of Case 4. (A) Diffusion-weighted image (DWI) at the level of the centrum semiovale demonstrating asymmetrically increased signal in the frontal cortex (red arrow) on the left with notable involvement of the cingulate gyrus bilaterally (red arrow). (B) T2-weighted fluid attenuation inversion recovery (T2-FLAIR) image with less corresponding hyperintense signal. (C) DWI at the level of the basal ganglia demonstrating asymmetrically increased signal in the striatum (red arrow), left insular cortex, and left superior temporal gyrus (yellow arrow). (D) T2-FLAIR image with less corresponding hyperintense signal.

The brain MRI demonstrated asymmetric restricted diffusion involving the striatum and frontal/temporal/insular cortex with notable involvement of the cingulate gyrus (Figure 4). Using the 2011 UCSF modified grading system, the imaging met criteria for “MRI definitely CJD.” There was no record of an EEG performed. A lumbar puncture was obtained demonstrating positive RT-QuIC, T-tau protein >4000 (positive), and positive 14-3-3 protein. The patient’s do-not-resuscitate (DNR) was signed the same day the lumbar puncture results returned, and the patient was discharged a few days later with hospice services (20 months after initial presentation).

Case 5

**Figure 5:**
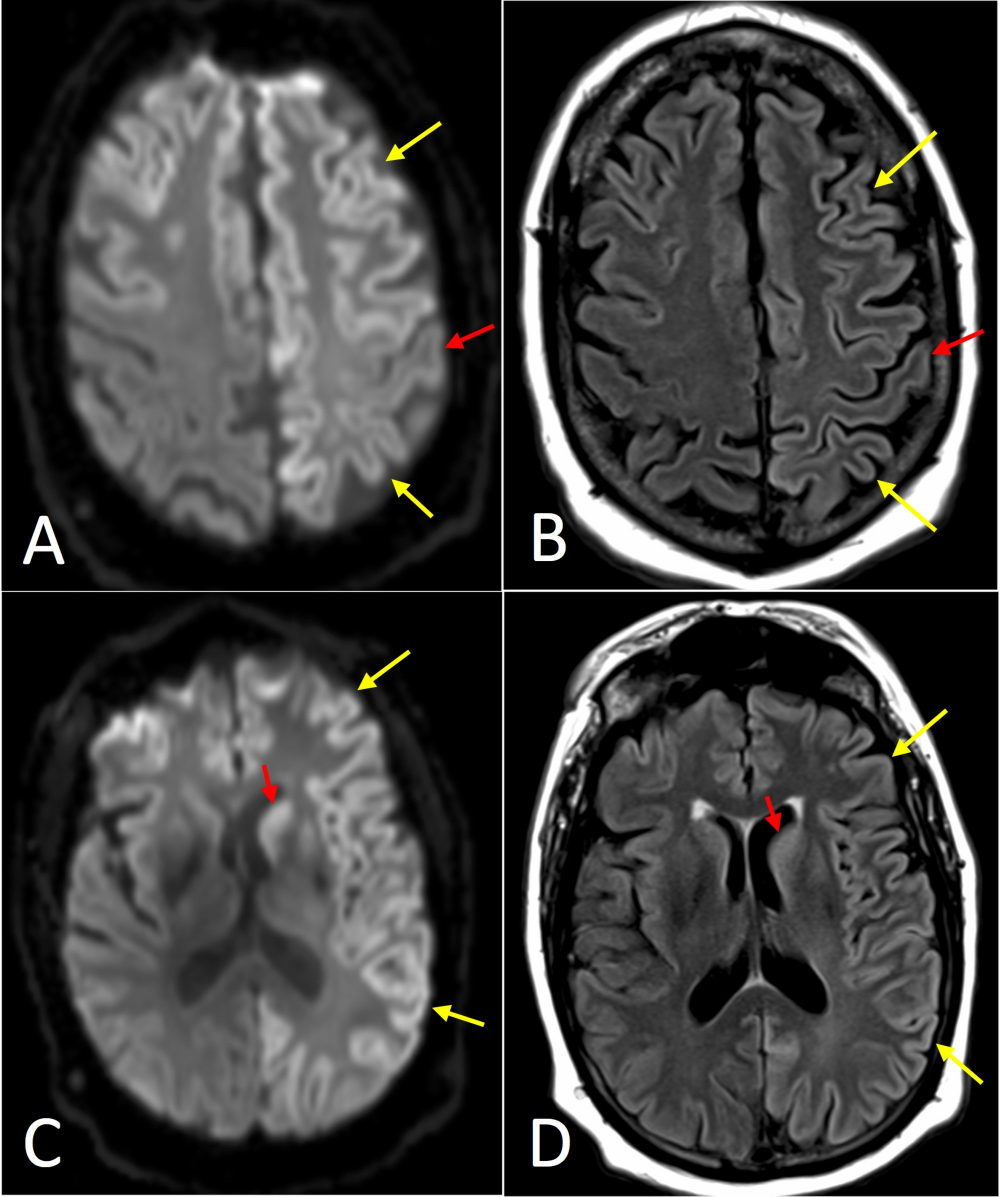
Axial magnetic resonance imaging (MRI) of Case 5. (A) Diffusion-weighted image (DWI) at the level of the centrum semiovale demonstrating asymmetrically increased signal in the frontal and parietal cortex (yellow arrows) on the left with relatively decreased signal in the precentral gyrus (red arrow). (B) T2-weighted fluid attenuation inversion recovery (T2-FLAIR) image with less corresponding hyperintense signal. (C) DWI at the level of the basal ganglia demonstrating asymmetrically increased signal in the neocortex (yellow arrow) and striatum (red arrow). (D) T2-FLAIR image with less corresponding hyperintense signal.

A 60-year-old male with a history of hemochromatosis, type 2 diabetes mellitus, and hypothyroidism presented to an outside hospital with word finding difficulty and blood glucose level of approximately 400 mg/dL (with HbA1c 13.1%). An MRI performed at the outside hospital was interpreted as only demonstrating age-related white matter changes. The patient experienced declining mental status with unintelligible speech two weeks after presentation and intermittent jerking and weakness of the right upper extremity. With continuing decline, the patient was transferred to our facility three weeks after initial presentation. A new MRI was performed at our facility, which demonstrated asymmetric (left greater than right), diffuse DWI hyperintensity of the bilateral cortex and striatum (Figure 5).

Using the 2011 UCSF modified grading system, the imaging met criteria for “MRI definitely CJD.” Results for CSF studies returned two weeks later and were negative for RT-QuIC, positive for T-tau, and inconclusive for 14-3-3 protein due to blood in the sample. An EEG showed generalized rhythmic delta activity with delta brush. Neonates normally may demonstrate a delta brush, which abates within the first month of life. Pathologically, the delta brush is a nonspecific sign of metabolic or structural abnormalities and often portends a worse prognosis in adult patients [6]. At this time the patient had a tracheostomy and would not follow any commands. The patient was transferred to hospice three months after the initial presentation.

## Discussion

CJD is classically described as a rapidly progressive dementia. Nonspecific symptoms cause difficulty in diagnosing CJD with top differential diagnoses including Alzheimer disease and dementia with Lewy bodies [3]. In addition to rapidly progressive dementia, at least two of the following clinical symptoms should be present to suggest CJD: myoclonus, visual abnormalities, pyramidal/extrapyramidal features, and akinetic mutism [8]. As seen in case 4, the patient was initially treated for Parkinson’s dementia. The age distribution typically includes the elderly population who are also more likely to have confounding neurological disorders, such as acute or chronic infarcts, as demonstrated in case 2. Many patients have movement disorders and gait difficulties, with myoclonic jerks being the most common [1]. While clinical presentation and physical examination cannot confirm CJD, it should be in the differential diagnosis for a patient presenting with rapidly progressive dementia.

CJD continues to present a diagnostic challenge with brain biopsy still considered the gold standard for diagnosis but carries the risk of contamination of the surgical suite and pathology lab. As demonstrated by the review of these five patients, clinical presentation, MRI, EEG, and CSF lab testing cannot always provide a definitive diagnosis. Table 2 summarizes the MRI, EEG, and laboratory findings. Fortunately, several studies have examined the diagnostic utility of EEG, MRI, and CSF testing both individually and in conjunction.

**Table 2:** A summary of the results for the five patient cases. *Blood in sample. MRI: Magnetic resonance imaging; UCSF: University of California-San Francisco; EEG: Electroencephalogram; RT-QuIC: Real-time quaking induced conversion; CJD: Creutzfeldt Jakob disease.

Case Number	MRI UCSF 2011 Diagnosis	EEG	RT-QuIC	T-tau	14-3-3 Protein
1	Definitely CJD	Suggestive	Positive	Positive	Positive
2	Definitely CJD	Suggestive	Positive	Positive	Positive
3	Definitely CJD	Normal	Positive	Negative	Negative
4	Definitely CJD	N/A	Positive	Positive	Positive
5	Definitely CJD	Suggestive	Negative	Positive	*Inconclusive

Imaging plays a vital role in the diagnosis of CJD. Computed tomography (CT) scans of the brain typically have little to no diagnostic value in the evaluation of rapidly progressive dementia, often demonstrating nonspecific atrophy or otherwise normal findings. MRI is the superior imaging test; specifically, DWI and T2-FLAIR. Spongiform changes propagate early in the disease process causing vacuole formation in the gray matter, which restricts diffusion with hyperintense signal greater on DWI than T2-FLAIR. Later in the disease process, gliosis predominates, and hyperintense signal on T2-FLAIR will typically be greater than on DWI [9].

Several guidelines have been created over the years to assist in diagnosing CJD including the World Health Organization, MRI-CJD consortium, and most recently the proposed UCSF revised criteria in 2011 [4]. Positive findings include hyperintensity on DWI greater than T2-FLAIR, with corresponding low ADC values, confirming restricted diffusion of water molecules. The 2011 UCSF criteria demonstrated a sensitivity of 98% and specificity of 100% for CJD. Typical areas with increased DWI signal are the neocortex, subcortical gray matter, and limbic system; however, limbic involvement alone does not occur with CJD but frequently occurs with other non-prion rapidly progressive dementias [4]. Another trial at a tertiary referral center for CJD found a sensitivity of 91% using MRI; however, the sensitivity from the initial referring centers was 47% [10]. The most reliable sign, hyperintense DWI signal in the cortex, was the finding most frequently missed at the initial referring centers. This demonstrates the need for increased awareness and recognition of characteristic MRI findings in CJD among all radiologists.

The five cases demonstrate the spectrum of typical MRI findings in CJD patients. Of patients with MRI findings consistent with CJD, only one-third have initial involvement of the basal ganglia [9]. It is suggested that cortical involvement precedes deep gray matter involvement, and patients with initial basal ganglia involvement typically display a more rapid disease progression. Sporadic CJD has several codon 129 polymorphisms, and the vast majority can be separated into six subgroups: MM1, MM2, MV1, MV2, VV1, and VV2 [3]. There are mixed results regarding the utility of MRI in differentiating these subgroups, but some patterns have been suggested [4, 11]. Two-thirds of cases are the MM1 and MV1 subtypes, which are generally more aggressive with a shorter disease course. These subtypes are more likely to have cortical and subcortical involvement. MM2 and VV1 subtypes generally have a less rapid disease course and sparing of the subcortical gray matter nuclei. The MM2 and VV1 subgroups often do not have typical EEG findings [9].

On EEG, sharp-wave complexes are the characteristic finding in CJD with a sensitivity and specificity of 66% and 74%, respectively [9]. The generalized spike wave complexes are periodic at 0.5 to 2 Hz in 60-80% of patients with CJD [1]. Even in our five cases, only two patients had EEG findings suggestive of CJD. EEG in case 3 was normal and the EEG of case 5 was performed at an outside facility and the report was unavailable. Case 4 began transitioning to hospice before EEG could be performed.

CSF testing allows for a less-invasive option to perform laboratory testing when compared to brain biopsy and pathologic analysis. RT-QuIC is a direct test for prion disease from CSF samples. RT-QuiC has demonstrated a sensitivity of 87-91% and specificity of 98-100% [7]. The 14-3-3 protein is a marker of neuronal damage and is not sensitive nor specific, but when combined with T-tau test, they have 79% sensitivity and 99% specificity [1]. The T-tau test measures the ratio of total tau to phosphorylated tau in the CSF.

## Conclusions

Radiological evaluation is a critical component of the initial workup for nonspecific neurodegenerative disorders because brain MRI may suggest the initial diagnosis of CJD, as demonstrated in our presented cases. Radiologist’s familiarity with the spectrum of classic MRI findings suggestive of sporadic CJD can facilitate early detection of this most common prion disease. Clinicians may also benefit from understanding the utility of the newer CSF laboratory studies (RT-QuiC, T-tau, and 14-3-3 protein), which are far less invasive than the gold standard of brain biopsy. Early diagnosis can help save medical resources, avoid unnecessary procedures, and guide clinicians to form appropriate plans of care with the patient and family.
